# Identification of molecular subtypes and a six-gene risk model related to cuproptosis for triple negative breast cancer

**DOI:** 10.3389/fgene.2022.1022236

**Published:** 2022-10-28

**Authors:** Baoxi Zhu, Songping Wang, Rui Wang, Xiaoliang Wang

**Affiliations:** Department of Thyroid and Breast Surgery, Anhui No.2 Provincial People’s Hospital,, Hefei, China

**Keywords:** triple negative breast cancer, cuproptosis, molecular subtypes, risk model, SIX-gene

## Abstract

**Background:** Breast cancer is the mostly diagnosed cancer worldwide, and triple negative breast cancer (TNBC) has the worst prognosis. Cuproptosis is a newly identified form of cell death, whose mechanism has not been fully explored in TNBC. This study thought to unveil the potential association between cuproptosis and TNBC.

**Materials and Methods:** Gene expression files with clinical data of TNBC downloaded from The Cancer Genome Atlas (TCGA) and Gene Expression Omnibus (GEO) databases were included in this study. Consensus clustering was utilized to perform molecular subtyping based on cuproptosis-associated genes. Limma package was applied to distinguish differentially expressed genes. Univariate Cox regression was used to identify prognostic genes. Least absolute shrinkage and selection operator and stepwise Akaike information criterion optimized and established a risk model.

**Results:** We constructed three molecular subtypes based on cuproptosis-associated genes, and the cuproptosis-based subtyping showed a robustness in different datasets. Clust2 showed the worst prognosis and immune-related pathways such as chemokine signaling pathway were significantly activated in clust2. Clust2 also exhibited a high possibility of immune escape to immune checkpoint blockade. In addition, a six-gene risk model was established manifesting a high AUC score over 0.85 in TCGA dataset. High- and low-risk groups had distinct prognosis and immune infiltration. Finally, a nomogram was constructed with strong performance in predicting TNBC prognosis than the staging system.

**Conclusion:** The molecular subtyping system related to cuproptosis had a potential in guiding immunotherapy for TNBC patients. Importantly, the six-gene risk model was effective and reliable to predict TNBC prognosis.

## Introduction

Breast cancer is one of the leading cause of cancer death in women, which is the top one diagnosed cancer type with 2,261,419 new cases (11.7% of total cases) in 2020 according to the global cancer statistics (Sung et al., 2021). The overall survival of breast cancer is markedly different in developed and developing countries, with an estimated 5-year survival of 80% and below 40%, respectively (Coleman et al., 2008). The incidence of breast cancer elevates with age but seldomly found before the age of 20 years and breast cancer most commonly occurs in 40–50 aged women (Akram et al., 2017). Although many versions of guidelines for the diagnosis and treatment of breast cancer have been established, such as European Breast Guidelines (Schünemann et al., 2020) and the American Joint Committee on Cancer’s (AJCC) guideline (Plichta et al., 2020), the treatment for triple negative breast cancer (TNBC) still remains a challenge. TNBC is a clinically aggressive type of breast cancer with poor survival, compared with other breast cancer types, including HER2-positive, oestrogen receptor (ER)-positive and progesterone receptor (PR)-positive. Chemotherapy resistance and immune escape common occur in TNBC, which makes an obstacle in TNBC treatment (Wein and Loi, 2017). Therefore, accurate molecular biomarkers or subtypes are strongly needed to guide personalized therapy for TNBC.

Programmed cell death is recognized as a promising therapeutic target in cancer therapy, where necroptosis, pyroptosis, and apoptosis are the most studied types (Bertheloot et al., 2021). Cuproptosis is considered as a new form of programmed cell death involved in the proliferation of lung cancer cells (Tang et al., 2022). Copper chelators such as RPTDH/R848 nanoparticles are demonstrated to be able to suppress cancer cell growth and metastasis in breast cancer (Zhou et al., 2019), inspiring a possibility that cuproptosis is a potential target for cancer treatment. Up to now, studies have discovered a series of prognostic signatures related to cuproptosis for different cancer types such as kidney renal clear cell carcinoma (Ji et al., 2022), melanoma (Lv et al., 2022), and hepatocellular carcinoma (Zhang et al., 2022). However, the relation between cuproptosis and TNBC has not been revealed.

Therefore, in this study, we aimed to analyze the role of cuproptosis in TNBC, and construct molecular subtypes based on cuproptosis-associated genes by using gene expression data of TNBC obtained from The Cancer Genome Atlas (TCGA) and Gene Expression Omnibus (GEO) databases. By comparing the molecular features of different subtypes, we unveiled the relation between cuproptosis and immune infiltration. Moreover, a risk model related to cuproptosis was established for predicting TNBC prognosis. The risk model was effective to distinguish TNBC patients into different risk types. Notably, the model outperformed the AJCC staging system, which had a potential to be used as a prognostic signature in TNBC.

## Materials and methods

### Data collection and preprocessing

The RNA-seq data of TNBC was downloaded from Genomic Data Commons (GDC) Data Portal by TCGA GDC API (https://portal.gdc.cancer.gov/projects/TCGA-BRCA, named as TCGA dataset). GSE103091 dataset was downloaded from GEO database (https://www.ncbi.nlm.nih.gov/geo/). TNBC samples without progression-free survival (PFS) or survival status were eliminated. TNBC samples with PFS shorter than 30 days or more than 10 years were excluded. In GSE103091 dataset, Ensembl ID was converted to gene symbol and we used the averaged expression level when a gene had multiple Ensembl IDs. Finally, 105 TNBC samples and 113 paracancerous samples were remained in TCGA dataset, and 91 TNBC samples were remained in GSE103091 dataset.

### The source of cuproptosis genes

Cuproptosis genes were obtained from a previous study (Tsvetkov et al., 2022), and a total of 13 cuproptosis genes were used in the study including FDX1, LIPT1, LIAS, DLD, DBT, GCSH, DLST, DLAT, PDHA1, PDHB, SLC31A1, ATP7A, and ATP7B.

### Identification of prognostic cuproptosis-associated genes

Firstly, single sample gene set enrichment anlaysis (ssGSEA) was used to calculate the enrichment score of 13 cuproptosis genes for each sample in TCGA dataset. Limma R package (Ritchie et al., 2015) was applied to screen differentially expressed genes (DEGs) between paracancerous and tumor samples (false discovery rate (FDR) < 0.05 and |log2FC| > 1). Then Pearson correlation analysis was performed to evaluate the correlation between the DEG expression and the ssGSEA score of cuproptosis. DEGs with |correlation coefficient (R)| > 0.4 and *p* < 0.05 were selected. Next, univariate Cox regression analysis in the survival R package was conducted on the DEGs and DEGs with *p* < 0.05 as the input for unsupervised consensus clustering.

### Constructing molecular subtypes based on prognostic cuproptosis-associated genes

ConsensusClusterPlus R package (Wilkerson and Hayes, 2010) was used for conducting unsupervised consensus clustering to identify molecular subtypes. The expression of prognostic cuproptosis-associated genes were used as a basis for clustering samples. KM algorithm and Euclidean distance were set to carry out 500 bootstraps with each bootstrap consisting of 80% of samples in TCGA dataset. Cluster number k was chosen from 2 to 10. The optimal cluster number was determined according to cumulative distribution function (CDF) and area under CDF curve.

### Gene set enrichment analysis

Gene set enrichment analysis (GSEA) (Subramanian et al., 2005) was utilized to calculate the enrichment score of functional pathways for molecular subtypes. Kyoto Encyclopedia of Genes and Genomes (KEGG) pathways were obtained from KEGG database (https://www.genome.jp/kegg/).

### Establishing a risk model

Firstly, DEGs between different molecular subtypes were identified with limma R package (FDR <0.05 and |log2FC| > 1.5). Least absolute shrinkage and selection operator (LASSO) regression analysis (Friedman et al., 2010) decreased the number of DEGs in glmnet R package. Stepwise Akaike information criterion (stepAIC) was applied for further optimizing the risk model through MASS R package (Zhang, 2016). We determined the risk model according to the formula: risk score=Σβi×Expi , where β indicates the coefficient of prognostic genes and Expi indicates the expression level of prognostic genes. Each sample obtained a risk score, which was subsequently transferred to z-score. Samples were stratified into high-risk and low-risk groups according to the z-score = 0. Kaplan-Meier survival analysis was conducted to evaluate the prognosis of the two risk groups.

### Assessment of immune infiltration

Estimation of STromal and Immune cells in MAlignant Tumours using Expression data (ESTIMATE) tool was implemented to evaluate stromal and immune infiltration (Yoshihara et al., 2013). Microenvironment Cell Populations (MCP)-counter methodology was applied to assess the enrichment of 10 immune cells (Becht et al., 2016). SsGSEA algorithm in GSVA R package was performed to predict estimated proportion of 28 immune cells (Hänzelmann et al., 2013).

### Statistical analysis

The bioinformatics analysis in this study was supported by Sangerbox platform (http://vip.sangerbox.com/) (Shen et al., 2022). R software (v4.1) was used as a platform to conduct all statistical analysis. Log-rank test was performed in Kaplan-Meier survival analysis, univariate and multivariate Cox regression analysis. Student t test was performed to examine the difference between two groups. ANOVA was conducted to test the difference among three groups. *p* < 0.05 was considered as statistically significant.

## Results

### Identification of prognostic genes associated with cuproptosis

**FIGURE 1:**
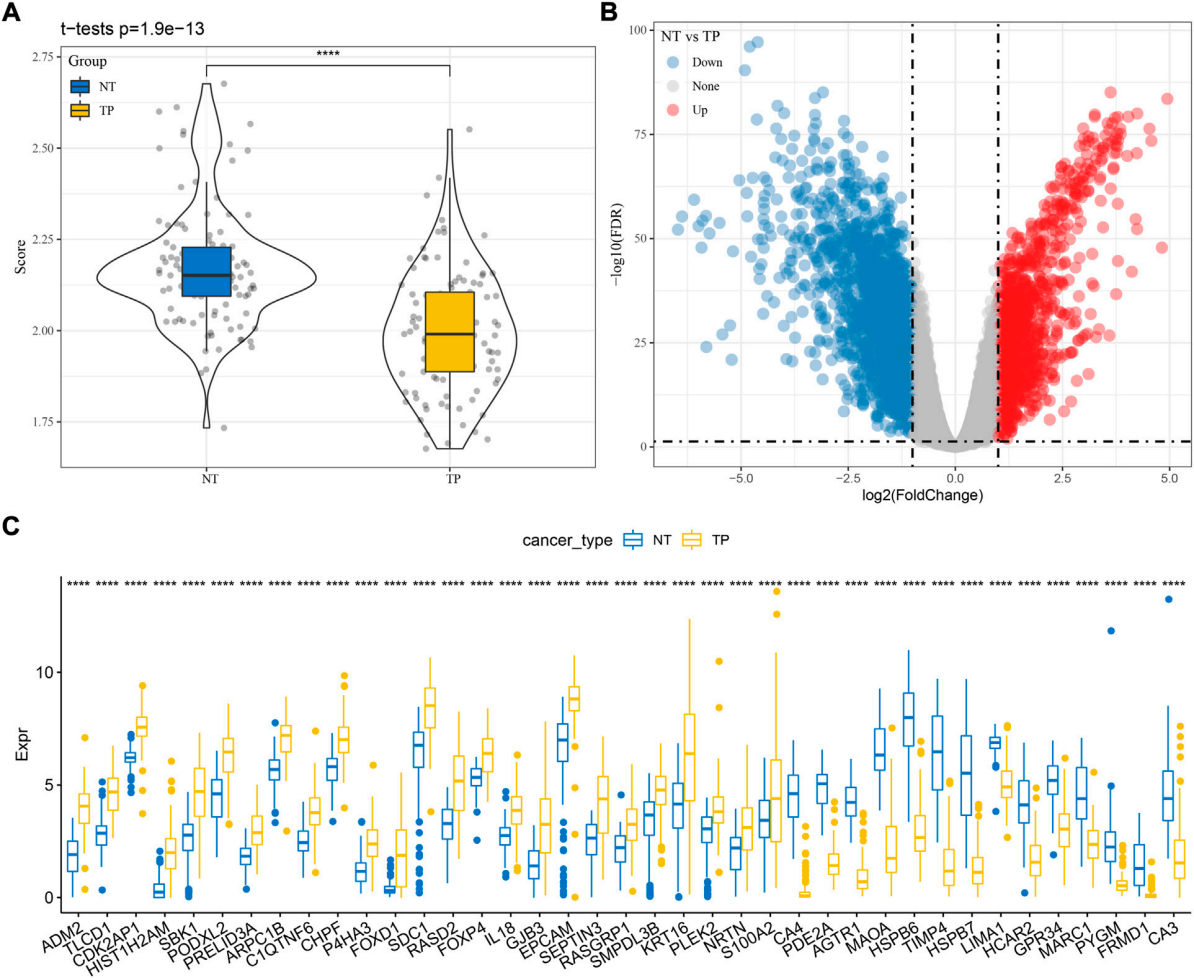
Identification of cuproptosis-associated genes related to TNBC prognosis in TCGA dataset. **(A)** The ssGSEA score of cuproptosis in paracancerous (NT) and tumor samples (TP). **(B)** Volcano plot of DEGs between NT and TP samples. **(C)** The expression of seven prognostic cuproptosis-associated genes in NT and TP samples. Student t test was performed. ****p < 0.0001.

Firstly, we calculated the ssGSEA score of cuproptosis pathway based on 13 cuproptosis genes for each TNBC sample in TCGA dataset (Supplementary Table S1). Paracancerous samples had obviously higher cuproptosis score than tumor samples (Figure 1A). Then differential analysis was performed to identify DEGs between TNBC and paracancerous samples. A total of 3125 DEGs were screened under FDR <0.05 and |log2FC| > 1 (Figure 1B). Next, we analyzed the relation between the expression of DEGs and ssGSEA of cuproptosis by Pearson correlation analysis. 1,275 DEGs with |R| > 0.4 and *p* < 0.05 were selected for further univariate Cox regression analysis (Supplementary Table S2). 39 prognostic DEGs were found to be significantly associated with TNBC prognosis in TCGA dataset (*p* < 0.05, Supplementary Table S3), whose expression levels were significantly different between paracancerous and tumor samples (*p* < 0.0001, Figure 1C).

### Construction of molecular subtypes based on cuproptosis-associated genes

**FIGURE 2:**
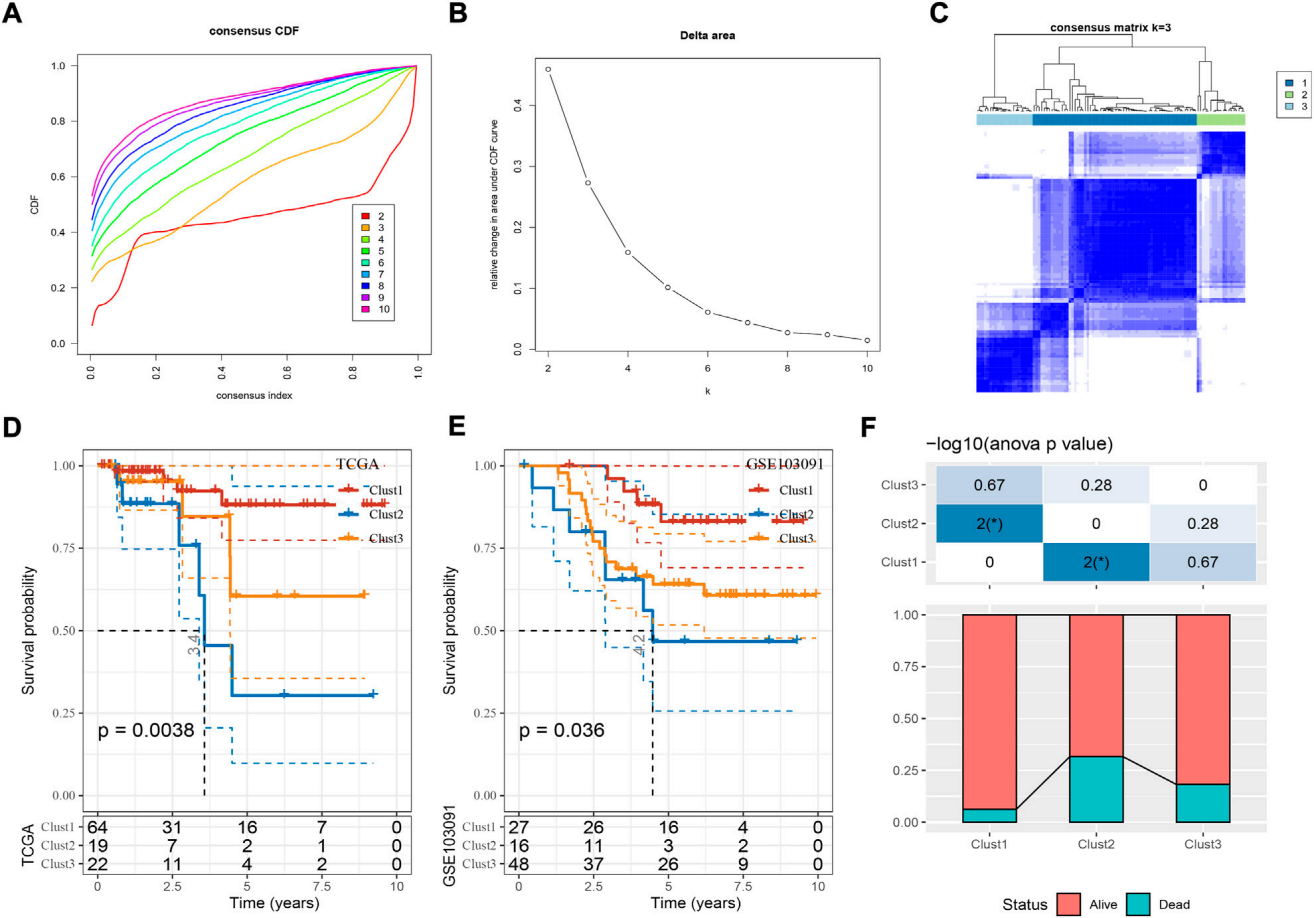
Construction of molecular subtypes based on cuproptosis-associated genes. **(A,B)** Consensus CDF curve and delta area under CDF curve when cluster number k = 2 to 10. **(C)** Consensus matrix when k = 3. **(D,E)** Kaplan-Meier survival curve of three molecular subtypes in TCGA **(D)** and GSE103091 **(E)** datasets. Log-rank test was performed. **(F)** The distribution of alive and dead samples in three subtypes. ANOVA was conducted. *p < 0.05.

Based on the expression profiles of the 39 cuproptosis-associated genes, we then constructed molecular subtypes through consensus clustering. According to the CDF curve, cluster number k = 3 was determined as the optimal (Figures 2A–C). Three molecular subtypes (clust1, clust2, and clust3) were distinguished based on the 39 cuproptosis-associated genes, and they showed distinct PFS in both TCGA and GSE103091 datasets (Figures 2D,E; Supplementary Figure S1, log-rank *p* = 0.0038 and 0.036, respectively). Clust2 had the shortest PFS and the most number of dead samples, while clust1 had the favorable prognosis (Figure 2F), indicating that cuproptosis-associated genes may be involved in the TNBC progression.

### Differential pathways and immune infiltration of three molecular subtypes

**FIGURE 3:**
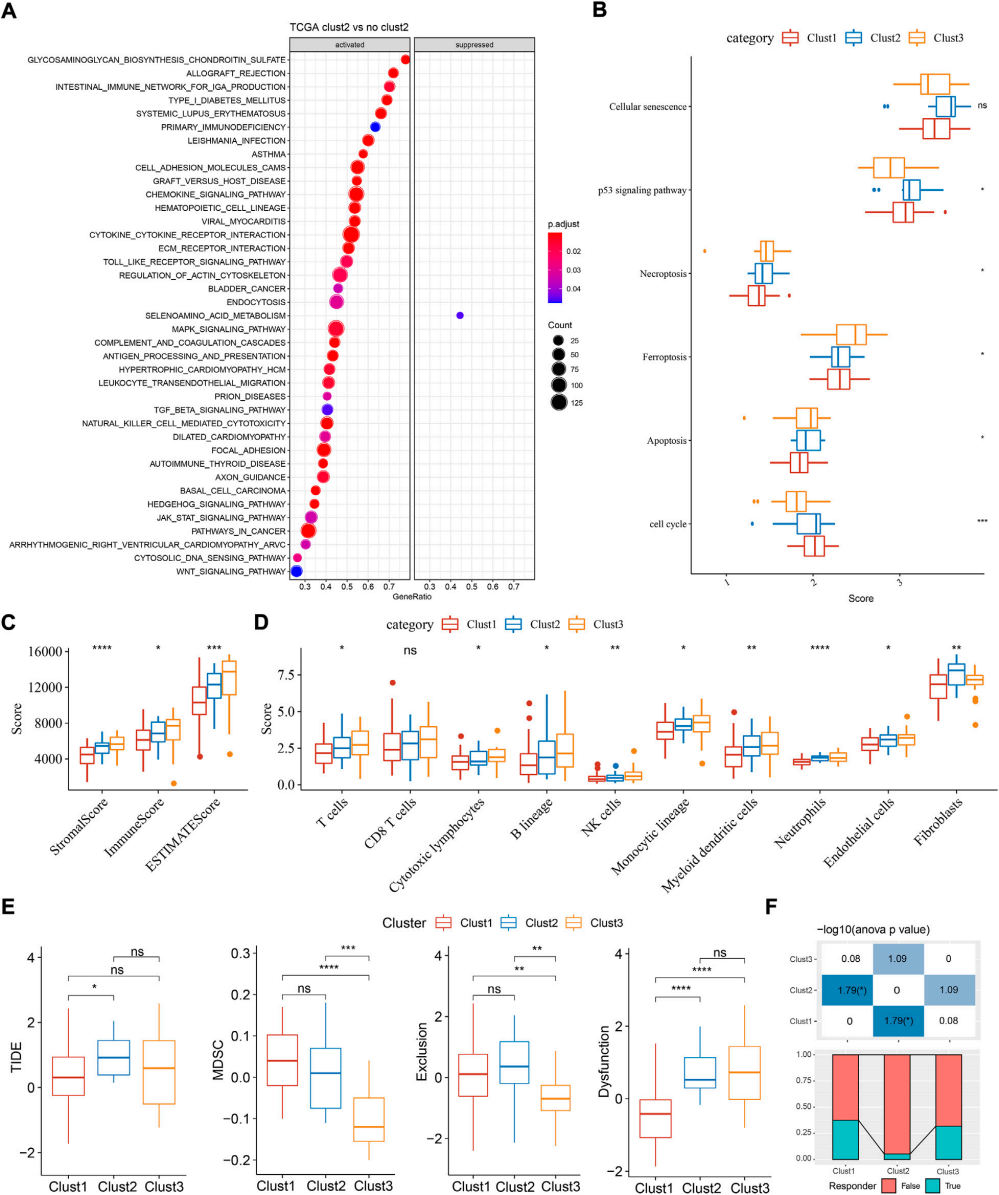
Functional analysis and immune analysis of three molecular subtypes in TCGA dataset. **(A)** GSEA result of clust2 vs non-clust2. **(B)** The ssGSEA score of six pathways related to cell death. **(C)** Stromal score and immune score calculated by ESTIMATE. **(D)** The estimated enrichment score of 10 immune cells. **(E)** TIDE analysis for predicting the sensitivity to immunotherapy. **(F)** The proportion of responders and non-responders in three subtypes. ANOVA was conducted. ns, not significant. *p < 0.05, **p < 0.01, ***p < 0.001, ****p < 0.0001.

Next we analyzed the enriched pathways of the three subtypes by GSEA. By comparing clust2 to non-clust2 (clust1 and clust3), we observed that immune-related pathways and tumor-related pathways were obviously activated in clust2, such as cytokine-cytokine receptor interaction, chemokine signaling pathway, MAPK signaling pathway, toll-like receptor signaling pathway, TGF-β signaling pathway, and pathways in cancer (Figure 3A). In clust1 vs non-clust1, the above pathways were significantly suppressed (Supplementary Figure S2), suggesting that cuproptosis-associated genes were involved in the immune regulation. Pathways related to cell proliferation and cell death were evaluated in the three subtypes. Among the six pathways, P53 signaling pathway was the most enriched in clust2 and clust1 had the lowest enrichment of cell death-related pathways including necroptosis, ferroptosis, and apoptosis (Figure 3B, ANOVA *p* < 0.05). This indicated an interaction of cuproptosis with other cell death pathways.

Given that immune-related pathways were differentially enriched in three subtypes, we then assessed the immune infiltration. Not surprisingly, clust1 had the lowest stromal score and immune score, compared with other two subtypes (Figure 2C, ANOVA *p* < 0.0001). Estimation of 10 immune cell types by MCP-counter also showed a lowest enrichment of them in clust1 such as T cells, monocytic lineage, and myeloid dendritic cells (*p* < 0.05, Figure 3D). Notably, clust2 had the highest enrichment of fibroblasts (*p* < 0.01, Figure 3D). Similar results were outputted through ssGSEA that majority of immune cells had a low estimated proportion in clust1 (Supplementary Figure S2B). Furthermore, we also determined the expression of immune checkpoint genes in the three subtypes. The result showed that 22 of 47 immune checkpoints were differentially expressed in the three subtypes (Supplementary Figure S2C). We suspected that cuproptosis-associated genes had an influence in tumor microenvironment and therefore affected the efficiency of immunotherapy in TNBC. TIDE analysis revealed the predicted sensitivity of three subtypes to immune checkpoint blockade therapy (Figure 3E). Clust2 had the highest TIDE score, suggesting a high possibility of immune escape to immunotherapy, which may be resulted from a high enrichment of myeloid-derived suppressor cells (MDSCs), T cell exclusion and T cell dysfunction (Figure 3E). The proportion of responders in clust2 was also the lowest compared with other two subtypes (Figure 3F).

### Construction of a cuproptosis-related risk model for predicting TNBC prognosis

As three subtypes performed different molecular signatures, we then identified the DEGs between clust1 vs non-clust1, clust2 vs non-clust2, clust3 vs non-clust3. As a result, 2,723 DEGs were screened (FDR <0.05 and |log2FC| > 1.5). Then univariate Cox regression was used to further filter 1,213 DEGs, and finally 89 DEGs (prognostic genes) with 77 risk genes and 12 protective genes remained (Supplementary Figure S3A). Moreover, LASSO regression was performed on 89 genes to generate an optimal risk model. The model reached the optimal when lambda = 0.057, where 14 prognostic genes remained (Supplementary Figure S3B, C). StepAIC was further performed to optimize the prognostic model, and finally six prognostic genes were remained including *PTPRN2*, *SCARB1*, *SLC37A2*, *YES1*, *LY6D*, and *NOTCH3* (Supplementary Figure S3D). The risk model was determined according to the following formula:

risk score=0.384*PTPRN2+(−0.754*SCARB1)+0.703*SLC37A2+(−0.586*YES1)+0.264*LY6D+0.622*NOTCH3

**FIGURE 4:**
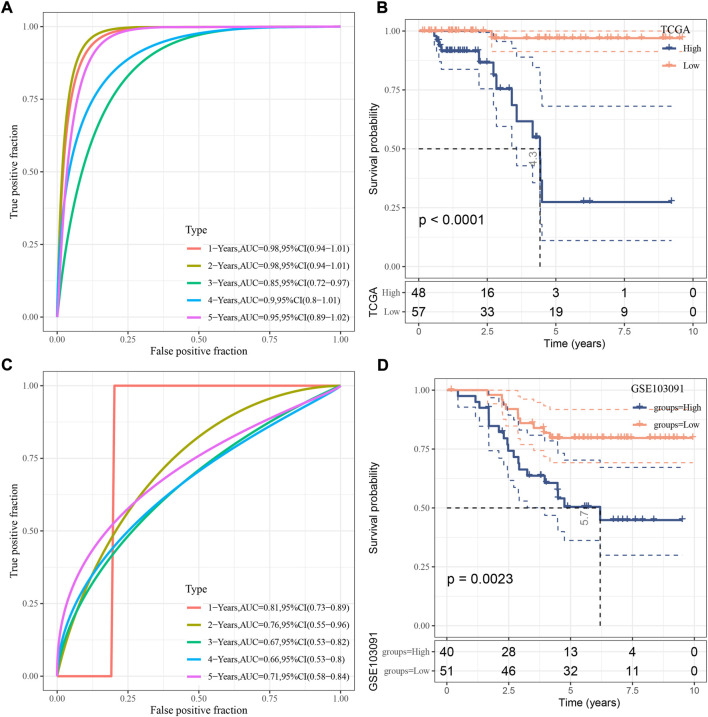
Verification of the risk model. **(A)** ROC analysis of the risk model in TCGA dataset. **(B)** Kaplan-Meier survival curve of two risk groups in TCGA dataset. **(C)** ROC analysis of the risk model in GSE103091 dataset. **(D)** Kaplan-Meier survival curve of two risk groups in GSE103091 dataset. Log-rank test was performed.

For each sample, a risk score was calculated according to the formula. The risk model showed a favorable performance in predicting one- to 5-year PFS with AUC all over than 0.85 in TCGA dataset (Figure 4A). Determined by the optimal cut-off value of risk score, the samples were classified to different risk types (high-risk and low-risk). Kaplan-Meier survival plot showed that high- and low-risk groups had markedly different PFS (Figure 4B, *p* < 0.0001). In GSE103091 dataset, a favorable AUC of the risk model and differential prognosis between two risk groups was also observed (Figures 4C,D).

### The association of risk score with clinical stages and immune infiltration

**FIGURE 5:**
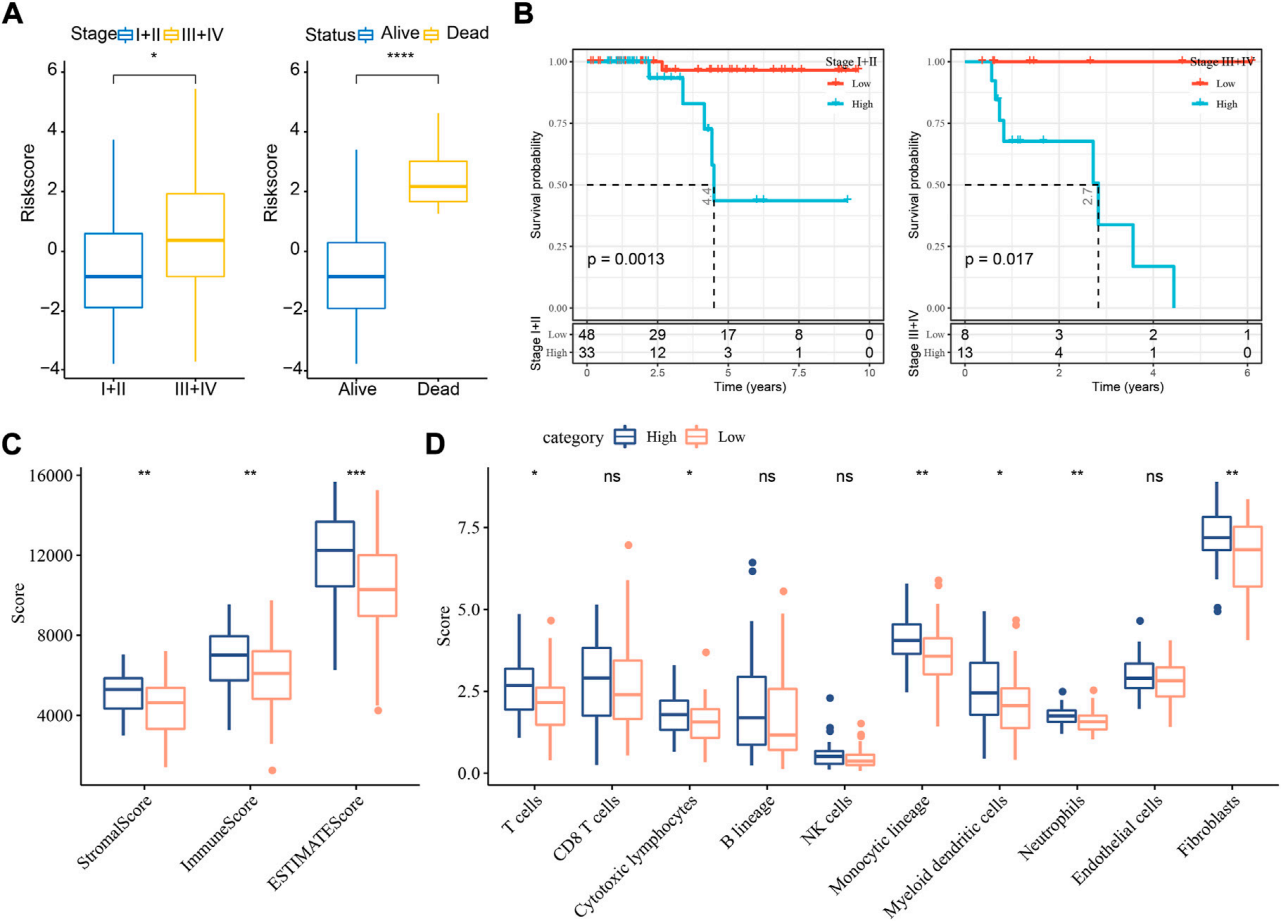
The relation of risk score to clinical features and immune infiltration in TCGA dataset. **(A)** The risk score in different stages and survival status. Student t test was conducted. **(B)** Kaplan-Meier survival analysis of high- and low-risk groups with different stages. Log-rank test was conducted. **(C)** ESTIMATE analysis for calculating stromal score and immune score of two groups. **(D)** MCP-counter analysis for calculating the enrichment score of 10 immune cells. Student t test was performed. ns, not significant. *p < 0.05, **p < 0.01, ***p < 0.001, ****p < 0.0001.

In the relation between risk score and clinical features, we found that a difference of risk score was shown between stage Ⅰ+Ⅱ and stage Ⅲ+Ⅳ (Figure 5A). In addition, alive samples had a lower risk score than the deceased samples. Kaplan-Meier survival analysis revealed that the risk model could effectively divide samples into high- and low-risk groups grouping by different clinical features (Figure 5B; Supplementary Figure S4A).

To understand whether a difference on tumor microenvironment was shown between two risk groups, we applied different tools, including ESTIMATE, MCP-counter, and ssGSEA, to evaluate the immune infiltration. The three tools showed consistent result that high immune infiltration was displayed in samples with high risk (Figures 5C,D, Supplementary Figure S4B). The above findings further demonstrated that cuproptosis-associated genes were possibly involved in the modulation of tumor microenvironment.

### Establishing a nomogram for clinical application based on risk score and clinical characteristics

**FIGURE 6:**
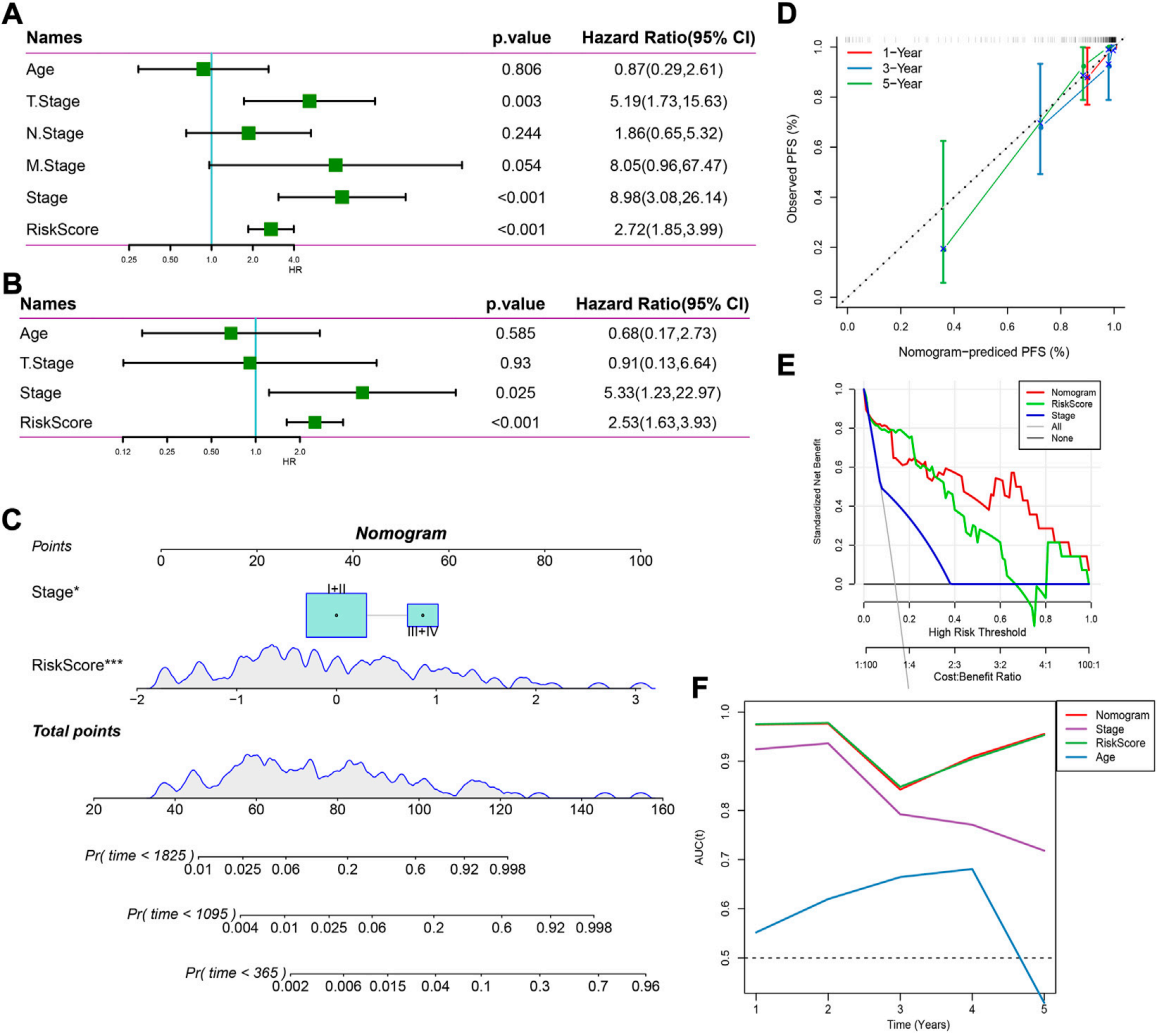
Establishing a nomogram based on the risk score. **(A,B)** Univariate **(A)** and multivariate **(B)** Cox regression analysis on age, stage, and risk score. **(C)** A nomogram for predicting death rate based on risk score and stage. **(D)** Calibration curve of 1-, 3-, and 5-year OS. **(E)** Decision curve of stage, risk score, and nomogram. **(F)** AUC of age, stage, risk score, and nomogram.

Univariate and multivariate Cox regression analysis revealed that stage and risk score were independent risk factors (Figures 6A,B). Consequently, we established a nomogram based on stage and risk score, of which risk score contributed the most to the nomogram (Figure 6C). Calibration curve showed that the predicted PFS was similar to the observed PFS (Figure 6D). Decision curve analysis (DCA) demonstrated the reliability of the nomogram and risk model (Figure 6E). Compared with other clinical characteristics, the nomogram and risk model exhibited a better performance in predicting PFS, especially long-term PFS (Figure 6F).

## Discussion

An increased level of copper can result in cell death and the disruption of cupper homeostasis can lead to life-threatening diseases such as Wilson’s disease and neurodegenerative disorders (Gaggelli et al., 2006; Bandmann et al., 2015). Tsvetkov et al. have revealed that copper-induced cell death, which defined as cuproptosis, is mediated by protein lipoylation involved in tricarboxylic acid (TCA) cycle (Tsvetkov et al., 2022). Unlike other cell death forms including apoptosis, ferroptosis, necroptosis, and pyroptosis, cuproptosis functions in a new mechanism through which mitochondrial ferredoxin 1-mediated protein lipoylation leads to proteotoxic stress and ultimately cell death (Tsvetkov et al., 2022). Cupper ionophores and cupper chelators have been explored as potential anti-cancer molecules (O'Day et al., 2013; Cui et al., 2021), which inspires the research on the potential of cuproptosis in cancer treatment.

We observed a significant difference of cuproptosis score between normal and TNBC samples, suggesting the instability of cuproptosis homeostasis in cancer cells. Normal samples have a higher cuproptosis score than TNBC samples, indicating a higher activity of cupper dwindling. Therefore, we further explored the association of cuproptosis with TNBC prognosis, functional pathways, and tumor immune microenvironment through constructing molecular subtypes based on cuproptosis-associated genes. The current study have shown that the three molecular subtypes had distinct prognosis and enrichment of activated pathways. Clust2 had the worst prognosis and the highest proportion of dead samples. Notably, immune related pathways were significantly activated in clust2, such as cytokine-cytokine signaling pathway, chemokine signaling pathway, and Toll-like receptor signaling pathway, which drove a possibility that cuproptosis may participate in the modulation of immune microenvironment. Not surprisingly, three molecular subtypes demonstrated different immune infiltration and response to immune checkpoint blockade. Clust2 was predicted to have a great possibility of immune escape in immunotherapy, compared to other two subtypes, which may be resulted from T cell exclusion and T cell dysfunction. Differential analysis on three molecular subtypes illustrated that cuproptosis was involved in cancer progression and immune microenvironment.

Furthermore, we established a risk model based on cuproptosis-related genes, where six prognostic biomarkers were included (PTPRN2, SCARB1, SLC37A2, YES1, LY6D, and NOTCH3). Most of these biomarkers have been reported to promote cancer progression. PTPRN2 is a protein tyrosine phosphatase receptor, which was found to be upregulated in metastatic breast cancer and could promote cancer metastasis through PI(4,5)P2-dependent actin remodeling (Sengelaub et al., 2016). Immature isoform of PTPRN2 (proPTPRN2) expression was closely associated with lymph node-positive breast cancer and poor clinical outcome (Sorokin et al., 2015). Scavenger receptor class B member 1 (SCARB1) is a cell-surface glycoprotein mediating low density lipoprotein-cholesteryl ester (LDL-CE), which is involved in lipid internalization (Swarnakar et al., 1999). David de Gonzalo-Calvo et al. suggested that SCARB1 potentially promote CE accumulation and aggressive feature in breast cancer (de Gonzalo-Calvo et al., 2015). Proto-oncogene tyrosine-protein kinase (YES1) has been widely reported to stimulate cancer cell growth and migration in various cancer types such as lung cancer (Garmendia et al., 2019), gastric cancer (Mao et al., 2021), and breast cancer (Takeda et al., 2017), which is therefore considered as a novel therapeutic target for cancer therapy (Garmendia et al., 2022). Targeting YES1 was effective to restore the sensitivity to chemotherapeutic drugs (trastuzumab and lapatinib) in drug-resistance breast cancer cell lines (Takeda et al., 2017). Moreover, downregulation of YES1 *via* miR-133 was demonstrated to inhibit cancer cell proliferation triple-negative breast cancer cell lines (Zhang et al., 2020). Lymphocyte antigen six superfamily member D (LY6D) has been identified as a biomarker for bladder cancer and a chemoresistance marker laryngeal squamous cell carcinoma (Andersson et al., 2020; Wang et al., 2020). NOTCH3 signaling is a well-known pathway contributing to cancer development (Aburjania et al., 2018). SLC37A2 has not been reported to be involved in cancerigenesis or cancer progression.

The risk model manifested a favorable performance in predicting TNBC prognosis in the two independent datasets. Two risk groups also showed different immune infiltration, which was consistent with the result on molecular subtypes. To increase the accuracy of the risk model in predicting TNBC prognosis, we further established a nomogram that exhibited a better performance than the staging system.

## Conclusion

In conclusion, this study revealed the important role of cuproptosis in TNBC development and its crosstalk with tumor immune microenvironment. We distinguished three molecular subtypes related to cuproprotiss, which had a potential to guide the personalized immunotherapy. In addition, we established a six-gene risk model with robust performance to predict TNBC prognosis.

## Data Availability

The datasets presented in this study can be found in online repositories. The names of the repository/repositories and accession number(s) can be found in the article/Supplementary Material.

## References

[B1] Aburjania Z. Jang S. Whitt J. Jaskula-Stzul R. Chen H. Rose J. B. (2018). The role of Notch3 in cancer. Oncologist 23, 900–911. 29622701 10.1634/theoncologist.2017-0677 PMC6156186

[B2] Akram M. Iqbal M. Daniyal M. Khan A. U. (2017). Awareness and current knowledge of breast cancer. Biol. Res. 50, 33. 28969709 10.1186/s40659-017-0140-9 PMC5625777

[B3] Andersson N. Ohlsson J. Wahlin S. Nodin B. Boman K. Lundgren S. (2020). Lymphocyte antigen 6 superfamily member D is A marker of urothelial and squamous differentiation: Implications for risk stratification of bladder cancer. Biomark. Res. 8, 51. 10.1186/s40364-020-00232-1 33042546 PMC7539380

[B4] Bandmann O. Weiss K. H. Kaler S. G. (2015). Wilson's disease and other neurological copper disorders. Lancet. Neurol. 14, 103–113. 10.1016/S1474-4422(14)70190-5 25496901 PMC4336199

[B5] Becht E. Giraldo N. A. Lacroix L. Buttard B. Elarouci N. Petitprez F. (2016). Estimating the population abundance of tissue-infiltrating immune and stromal cell Populations using gene expression. Genome Biol. 17, 218. 10.1186/s13059-016-1070-5 27765066 PMC5073889

[B6] Bertheloot D. Latz E. Franklin B. S. (2021). Necroptosis, pyroptosis and apoptosis: An intricate game of cell death. Cell. Mol. Immunol. 18, 1106–1121. 10.1038/s41423-020-00630-3 33785842 PMC8008022

[B7] Coleman M. P. Quaresma M. Berrino F. Lutz J. M. De Angelis R. Capocaccia R. (2008). Cancer survival in five continents: A worldwide population-based study (concord). Lancet. Oncol. 9, 730–756. 10.1016/S1470-2045(08)70179-7 18639491

[B8] Cui L. Gouw A. M. Lagory E. L. Guo S. Attarwala N. Tang Y. (2021). Mitochondrial copper depletion suppresses triple-negative breast cancer in mice. Nat. Biotechnol. 39, 357–367. 10.1038/s41587-020-0707-9 33077961 PMC7956242

[B9] De Gonzalo-Calvo D. López-Vilaró L. Nasarre L. Perez-Olabarria M. Vázquez T. Escuin D. (2015). Intratumor cholesteryl ester accumulation is associated with human breast cancer proliferation and aggressive potential: A molecular and clinicopathological study. Bmc Cancer 15, 460. 10.1186/s12885-015-1469-5 26055977 PMC4460760

[B10] Friedman J. Hastie T. Tibshirani R. (2010). Regularization paths for generalized linear models via coordinate descent. J. Stat. Softw. 33, 1–22. 10.18637/jss.v033.i01 20808728 PMC2929880

[B11] Gaggelli E. Kozlowski H. Valensin D. Valensin G. (2006). Copper homeostasis and neurodegenerative disorders (Alzheimer's, Prion, And Parkinson's Diseases And Amyotrophic Lateral Sclerosis). Chem. Rev. 106, 1995–2044. 10.1021/cr040410w 16771441

[B12] Garmendia I. Pajares M. J. Hermida-Prado F. Ajona D. Bértolo C. Sainz C. (2019). Yes1 Drives Lung Cancer Growth And Progression And Predicts Sensitivity To Dasatinib. Am. J. Respir. Crit. Care Med. 200, 888–899. 10.1164/rccm.201807-1292OC 31166114

[B13] Garmendia I. Redin E. Montuenga L. M. Calvo A. (2022). Yes1: A Novel Therapeutic Target And Biomarker In Cancer. Mol. Cancer Ther. 21, 1371–1380. 10.1158/1535-7163.MCT-21-0958 35732509

[B14] Hänzelmann S. Castelo R. Guinney J. (2013). Gsva: Gene Set Variation Analysis For Microarray And Rna-Seq Data. Bmc Bioinforma. 14, 7. 10.1186/1471-2105-14-7 PMC3618321 23323831

[B15] Ji Z. H. Ren W. Z. Wang H. Q. Gao W. Yuan B. (2022). Molecular Subtyping Based On Cuproptosis-Related Genes And Characterization Of Tumor Microenvironment Infiltration In Kidney Renal Clear Cell Carcinoma. Front. Oncol. 12, 919083. 10.3389/fonc.2022.919083 35875087 PMC9299088

[B16] Lv H. Liu X. Zeng X. Liu Y. Zhang C. Zhang Q. (2022). Comprehensive Analysis Of Cuproptosis-Related Genes In Immune Infiltration And Prognosis In Melanoma. Front. Pharmacol. 13, 930041. 10.3389/fphar.2022.930041 35837286 PMC9273972

[B17] Mao L. Yuan W. Cai K. Lai C. Huang C. Xu Y. (2021). Epha2-Yes1-Anxa2 Pathway Promotes Gastric Cancer Progression And Metastasis. Oncogene 40, 3610–3623. 10.1038/s41388-021-01786-6 33941853 PMC8134040

[B18] O'day S. J. Eggermont A. M. Chiarion-Sileni V. Kefford R. Grob J. J. Mortier L. (2013). Final Results Of Phase Iii Symmetry Study: Randomized, Double-Blind Trial Of Elesclomol Plus Paclitaxel Versus Paclitaxel Alone As Treatment For Chemotherapy-Naive Patients With Advanced Melanoma. J. Clin. Oncol. 31, 1211–1218. 10.1200/JCO.2012.44.5585 23401447

[B19] Plichta J. K. Ren Y. Thomas S. M. Greenup R. A. Fayanju O. M. Rosenberger L. H. (2020). Implications For Breast Cancer Restaging Based On The 8th Edition Ajcc Staging Manual. Ann. Surg. 271, 169–176. 10.1097/SLA.0000000000003071 30312199 PMC6588495

[B20] Ritchie M. E. Phipson B. Wu D. Hu Y. Law C. W. Shi W. (2015). Limma Powers Differential Expression Analyses For Rna-Sequencing And Microarray Studies. Nucleic Acids Res. 43, E47. 10.1093/nar/gkv007 25605792 PMC4402510

[B21] Schünemann H. J. Lerda D. Quinn C. Follmann M. Alonso-Coello P. Rossi P. G. (2020). Breast Cancer Screening And Diagnosis: A Synopsis Of The European Breast Guidelines. Ann. Intern. Med. 172, 46–56. 10.7326/M19-2125 31766052

[B22] Sengelaub C. A. Navrazhina K. Ross J. B. Halberg N. Tavazoie S. F. (2016). Ptprn2 And Plcβ1 Promote Metastatic Breast Cancer Cell Migration Through Pi(4, 5)P2-Dependent Actin Remodeling. Embo J. 35, 62–76. 10.15252/embj.201591973 26620550 PMC4717998

[B23] Shen W. Song Z. Xiao Z. Huang M. Shen D. Gao P. (2022). Sangerbox: A Comprehensive, Interaction‐Friendly Clinical Bioinformatics Analysis Platform. Imeta 1. 10.1002/Imt2.36 PMC10989974 38868713

[B24] Sorokin A. V. Nair B. C. Wei Y. Aziz K. E. Evdokimova V. Hung M. C. (2015). Aberrant Expression Of Proptprn2 In Cancer Cells Confers Resistance To Apoptosis. Cancer Res. 75, 1846–1858. 10.1158/0008-5472.CAN-14-2718 25877877 PMC4417433

[B25] Subramanian A. Tamayo P. Mootha V. K. Mukherjee S. Ebert B. L. Gillette M. A. (2005). Gene Set Enrichment Analysis: A Knowledge-Based Approach For Interpreting Genome-Wide Expression Profiles. Proc. Natl. Acad. Sci. U. S. A. 102, 15545–15550. 10.1073/pnas.0506580102 16199517 PMC1239896

[B26] Sung H. Ferlay J. Siegel R. L. Laversanne M. Soerjomataram I. Jemal A. (2021). Global Cancer Statistics 2020: Globocan Estimates Of Incidence And Mortality Worldwide For 36 Cancers In 185 Countries. Ca. Cancer J. Clin. 71, 209–249. 10.3322/caac.21660 33538338

[B27] Swarnakar S. Temel R. E. Connelly M. A. Azhar S. Williams D. L. (1999). Scavenger Receptor Class B, Type I, Mediates Selective Uptake Of Low Density Lipoprotein Cholesteryl Ester. J. Biol. Chem. 274, 29733–29739. 10.1074/jbc.274.42.29733 10514447

[B28] Takeda T. Yamamoto H. Kanzaki H. Suzawa K. Yoshioka T. Tomida S. (2017). Yes1 Signaling Mediates The Resistance To Trastuzumab/Lap Atinib In Breast Cancer. Plos One 12, E0171356. 10.1371/journal.pone.0171356 28158234 PMC5291431

[B29] Tang D. Chen X. Kroemer G. (2022). Cuproptosis: A Copper-Triggered Modality Of Mitochondrial Cell Death. Cell Res. 32, 417–418. 10.1038/s41422-022-00653-7 35354936 PMC9061796

[B30] Tsvetkov P. Coy S. Petrova B. Dreishpoon M. Verma A. Abdusamad M. (2022). Copper Induces Cell Death By Targeting Lipoylated Tca Cycle Proteins. Science 375, 1254–1261. 10.1126/science.abf0529 35298263 PMC9273333

[B31] Wang J. Fan J. Gao W. Wu Y. Zhao Q. Chen B. (2020). Ly6d As A Chemoresistance Marker Gene And Therapeutic Target For Laryngeal Squamous Cell Carcinoma. Stem Cells Dev. 29, 774–785. 10.1089/scd.2019.0210 32178572

[B32] Wein L. Loi S. (2017). Mechanisms Of Resistance Of Chemotherapy In Early-Stage Triple Negative Breast Cancer (Tnbc). Breast 34, S27–S30. 10.1016/j.breast.2017.06.023 28668293

[B33] Wilkerson M. D. Hayes D. N. (2010). Consensusclusterplus: A Class Discovery Tool With Confidence Assessments And Item Tracking. Bioinformatics 26, 1572–1573. 10.1093/bioinformatics/btq170 20427518 PMC2881355

[B34] Yoshihara K. Shahmoradgoli M. Martínez E. Vegesna R. Kim H. Torres-Garcia W. (2013). Inferring Tumour Purity And Stromal And Immune Cell Admixture From Expression Data. Nat. Commun. 4, 2612. 10.1038/ncomms3612 24113773 PMC3826632

[B35] Zhang G. Sun J. Zhang X. (2022). A Novel Cuproptosis-Related Lncrna Signature To Predict Prognosis In Hepatocellular Carcinoma. Sci. Rep. 12, 11325. 10.1038/s41598-022-15251-1 35790864 PMC9256635

[B36] Zhang G. Wang J. Zheng R. Song B. Huang L. Liu Y. (2020). Mir-133 Targets Yes1 And Inhibits The Growth Of Triple-Negative Breast Cancer Cells. Technol. Cancer Res. Treat. 19, 1533033820927011. 10.1177/1533033820927011 32462982 PMC7278099

[B37] Zhang Z. (2016). Variable Selection With Stepwise And Best Subset Approaches. Ann. Transl. Med. 4, 136. 10.21037/atm.2016.03.35 27162786 PMC4842399

[B38] Zhou P. Qin J. Zhou C. Wan G. Liu Y. Zhang M. (2019). Multifunctional Nanoparticles Based On A Polymeric Copper Chelator For Combination Treatment Of Metastatic Breast Cancer. Biomaterials 195, 86–99. 10.1016/j.biomaterials.2019.01.007 30623789

