# Supplementary figures and images for: Identification of molecular subtypes and a six-gene risk model related to cuproptosis for triple negative breast cancer
Source: Front Genet. 2022 Oct 28;13:1022236. doi: 10.3389/fgene.2022.1022236 (PMC9649643; doi:10.3389/fgene.2022.1022236)

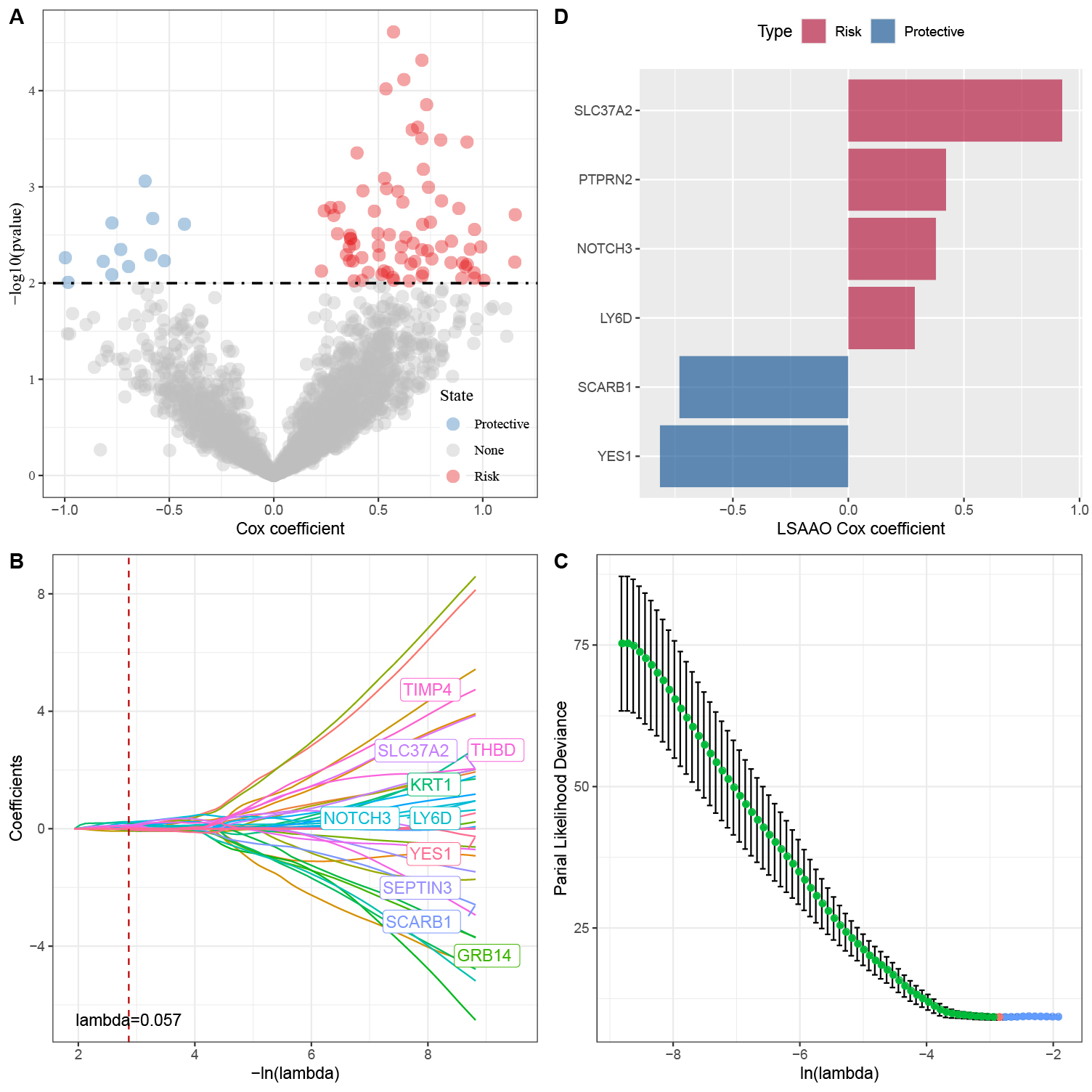

Supplement: Supplementary file 1 [file Image3.JPEG]

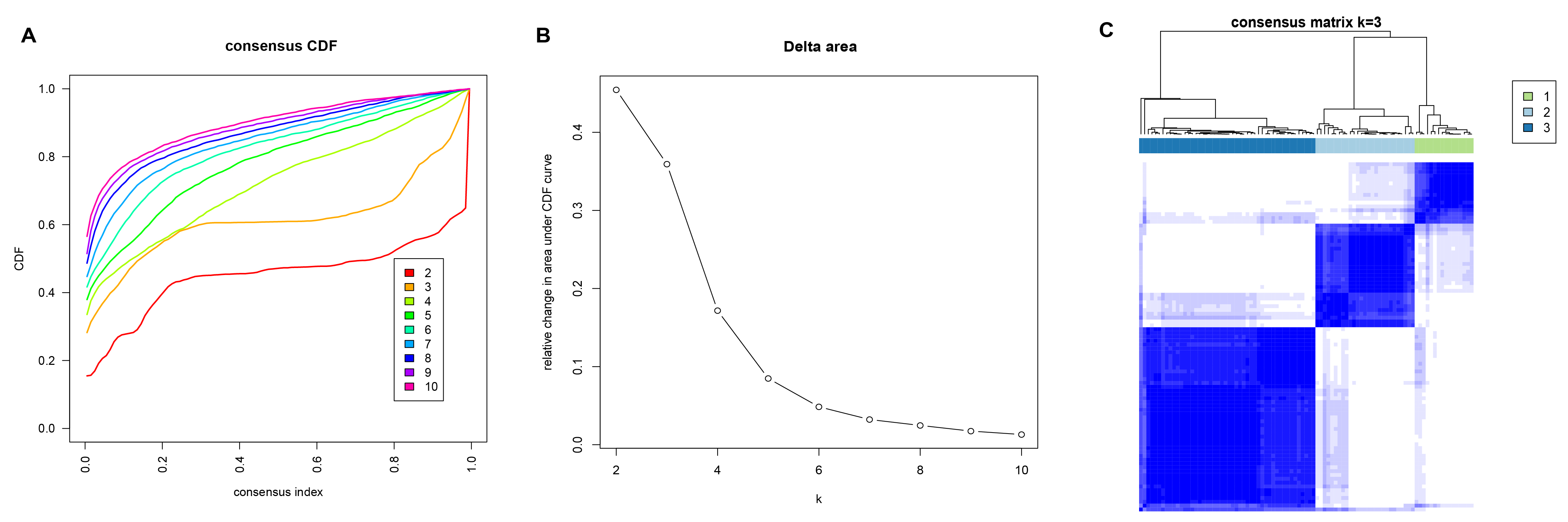

Supplement: Supplementary file 4 [file Image1.JPEG]

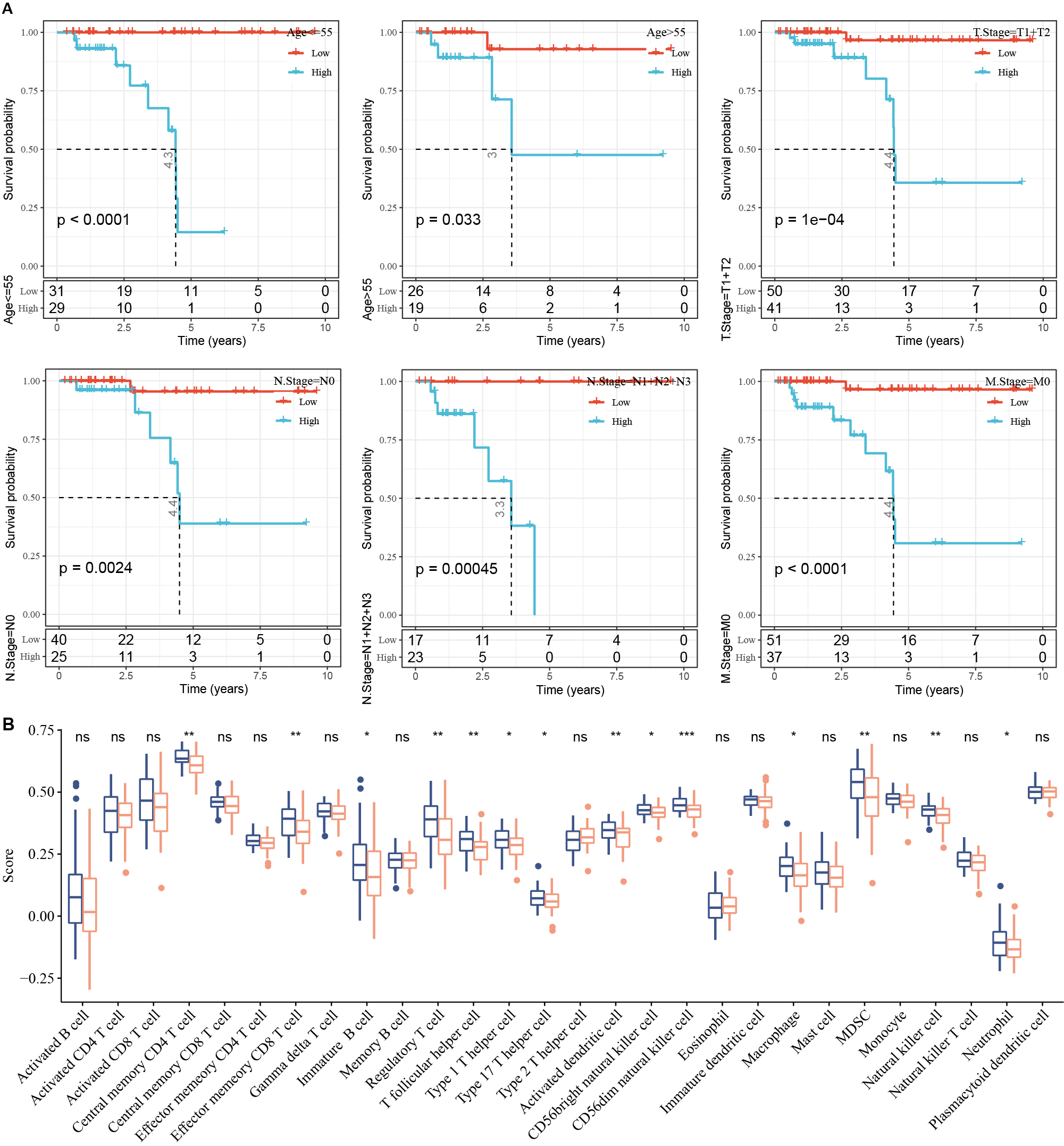

Supplement: Supplementary file 5 [file Image4.JPEG]

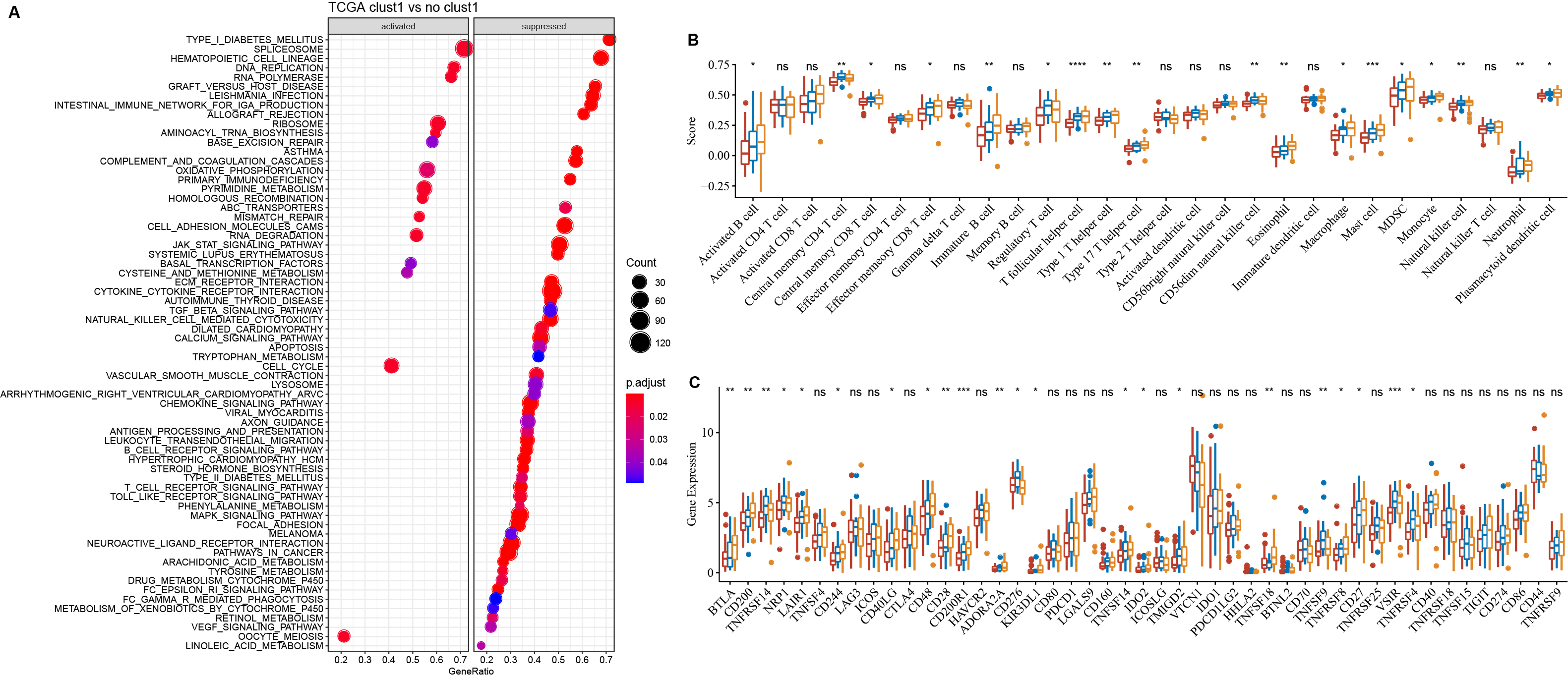

Supplement: Supplementary file 6 [file Image2.JPEG]
