# Supplementary material for: Identification of molecular subtypes and a six-gene risk model related to cuproptosis for triple negative breast cancer
Source: Front Genet. 2022 Oct 28;13:1022236. doi: 10.3389/fgene.2022.1022236 (PMC9649643; doi:10.3389/fgene.2022.1022236)
Supplement: Supplementary file 3 [file Table1.DOCX]

There is the data analyzed in this study:

https://www.jianguoyun.com/p/Dc0Eh88QharcChjMzdEEIAA
