# Supplementary material for: Identification of molecular subtypes and a six-gene risk model related to cuproptosis for triple negative breast cancer
Source: Front Genet. 2022 Oct 28;13:1022236. doi: 10.3389/fgene.2022.1022236 (PMC9649643; doi:10.3389/fgene.2022.1022236)
Supplement: Supplementary file 7 [file Table2.DOCX]

Supplementary materials

Figure S1. Consensus clustering of samples in GSE103091 dataset based on cuproptosis-related genes. (A-B) Consensus CDF curve and delta area under CDF curve when cluster number k = 2 to 10. (C) Consensus matrix when k = 3.

Figure S2. (A) GSEA of clust1 vs. non-clust1 in TCGA dataset. (B) SsGSEA score of 28 immune cells in three subtypes of TCGA dataset. (C) The expression of immune checkpoint genes in three subtypes of TCGA dataset.

Figure S3. Identification of 10 prognostic genes. (A) Volcano plot showing the 89 prognostic DEGs. (B) LASSO regression analysis on 39 prognostic genes. The coefficients of prognostic genes close to zero with the increasing lambda value. Red dashed line indicates lambda = 0.057. (C) Partial likelihood deviance of lambda value. (D) The six prognostic genes for constructing the risk model.

Figure S4. (A) Kaplan-Meier survival analysis of high- and low-risk groups with different clinical features. Log-rank test was performed. (B) The enrichment score of 28 immune cells. ns, not significant. *P < 0.05, **P < 0.01.

Table S1. The ssGSEA score of cuproptosis metabolism in TCGA dataset

Table S2. A list of 1275 DEGs significantly correlated with cuproptosis metabolism

Table S3. A list of 39 prognostic DEGs
